# Prognostic differences between VAP from *Acinetobacter baumanii* and VAP from other microorganisms

**Published:** 2012-04-30

**Authors:** Marianna Di Bonito, Simona Caiazzo, Marta Iannazzone, Viviana Miccichè, Giuseppe De Marco, Edoardo De Robertis, Rosalba Tufano, Ornella Piazza

**Affiliations:** 1 Department of Anesthesia and Intensive Care, Federico II University, Napoli, Italy; 2 Department of Medicine, University of Salerno, Salerno, Italy

**Keywords:** Acinetobacter baumanii, VAP, Colistin

## Abstract

Nosocomial infection, in particular pneumonia, is an important risk factor for hospital mortality and morbidity. *Acinetobacter baumanii* is a common multi-resistant microorganism responsible of Ventilator Associated Pneumonia (VAP). Currently Colistin is a rescue therapy for this pathogen. The purpose of this retrospective study is to compare the outcome of VAP caused by *Acinetobacter baumanii* and VAP from other microorganisms in critical patients. Comorbidity, prognostic scores, mortality and eradication frequency did not turn out significantly different between the two study groups. Colistin safety was tested.

## I. INTRODUCTION

A nosocomial infection, also known as a hospital-acquired infection or HAI, is an infection whose development is favored by a hospital environment, such as one acquired by a patient during a hospital visit or one developing among hospital staff. Such infections include fungal and bacterial infections and are aggravated by the reduced resistance of individual patients [1].

In the United States, the Centers for Disease Control and Prevention estimate that roughly 1.7 million hospital-associated infections, from all types of microorganisms, including bacteria, combined, cause or contribute to 99,000 deaths each year [2]. In European hospital surveys, the category of Gram-negative infections is estimated to account for two-thirds of the 25,000 deaths each year. Nosocomial infections can cause severe pneumonia and infections of the urinary tract, bloodstream and other parts of the body.

Many HAI are difficult to fight against with antibiotics and antibiotic resistance is spreading among Gram-negative bacteria that can infect people outside the hospital [3, 4].

VAP refers to a type of pneumonia occurring more than 48–72 hours after endotracheal intubation. It is the most common nosocomial infection in the intensive care unit (ICU) with an incidence of 10–20% out of all mechanically ventilated patients. Its prevalence increases with the duration of mechanical ventilation. VAP also increases the risk of all-cause mortality by 2–2.5 times, compared to patients without VAP[5]. The crude mortality from VAP has been reported to be 20–70%, and the mortality related could be as high as 50%. The development of VAP is associated with longer duration of mechanical ventilation, ICU stay, hospital stay and higher costs.

Among the microorganism involved in the pathogenesis of VAP, *Pseudomonas aeruginosa* and *Acinetobacter baumanii* are the predominant species and, together with Methicillin-resistant *Staphylococcus aureus*, account for the highest related mortality [5].

*Acinetobacter baumanii* is a non-fermenting, Gram-negative, aerobic coccobacillus found extensively in natural environments that has emerged as one of the most troublesome pathogens for health care institutions globally [6, 7, 8]. This microorganism is characterized by the rapid development of resistance to the majority of antimicrobials, including aminoglycosides, fluoroquinolones, and carbapenems [9,10,11,12,13,14]. In addition from its resistance profile, *A. baumanii* shows the ability to survive for prolonged periods throughout a hospital environment, thus potentiating its ability for nosocomial spread. As reported by reviews dating back to the 1970s, hospital acquired pneumonia is still the most common infection caused by this organism. However, more recently, infections involving the central nervous system, skin and soft tissue, and bone have emerged as highly problematic for certain institutions [15,16, 17,18,19,20].

The emergence of *A. baumannii* strains resistant to all routinely tested antimicrobials has led to the necessary revival of Colistin, a polypeptide antibiotic also known as polymyxin E (limited in use because of its neuro- and nephrotoxicity). This positively charged antimicrobial peptide was discovered in 1947, originating from Bacillus polymyxa. It is thought to target the anionic LPS molecules in the outer cell membranes of Gram-negative bacteria, leading to interactions between the inner and outer cell membranes, with associated lipid exchange, membrane disturbance, osmotic instability and eventual cell death [21].

In vitro Colistin has a broad spectrum of action against Gram-negative bacteria, including some strains resistant to penicillins, carbapenems, aminoglycosides and fluoroquinolones. However, *Proteus mirabilis*, *Providencia* spp., *Serratia* spp., *Burkholderia cepacia* and *Stenotrophomonas maltophilia* are naturally not susceptible to Colistin. There are two commercially available forms of Colistin: Colistin sulfate for oral and topical use and CMS for parenteral use (also known as sodium Colistin methanesulfonate or Colistin sulfomethate sodium) [22].

Despite its extensive use against *A. baumanii*, Colistin-resistant outbreaks are quite sporadic when compared with the widespread dissemination of resistance for other antibiotics, such as carbapenems. Recently, variation in the levels of 35 proteins between *A. baumannii* American Type Culture Collection (ATCC) strain 19606 and its in-vitro-derived Colistin-resistant mutant, RC64 (Colistin minimal inhibitory concentration, 2 and 64 lg/mL, respectively), has been shown by differential proteomics. This included changes in the expression of outer membrane proteins, chaperones, translation factors, and metabolic enzymes in RC64. In addition, this strain showed impaired in vitro fitness and longer duplication times compared with the parental strain. Colistin resistance in Gram-negative bacteria is most commonly due to decreased binding to the bacterial outer membrane [23, 24].

The aim of this study was to find the incidence, mortality and etiology of VAP, Colistin efficacy and patients outcome to formulate an institutional antimicrobial policy. Such an approach of guided empirical antibiotic therapy has shown to decrease morbidity and mortality, duration of treatment and hospital stay, and has also helped reduce costs and prevent development of multi-drug resistant strains.

## II. METHODOLOGY

### Study population

This study was performed at the ICU of AOUP “Federico II”, a medical surgical ten-bed unit. We performed a retrospective analysis of prospectively collected data on all patients admitted to our ICU from 1 January 2011 to 1 January 2012. The following data were collected for all patients: age, sex, risk factors, chest XR (CXR), body temperature, leukocyte count, creatinine, presence of sputum, microbiological results of non-bronchoscopic bronchoalveolar lavage (BAL), predicted mortality rate based on the Simplified Acute Physiology II Score (SAPSII), Sequential Organ Failure Assessment (SOFA) score at admission, SOFA at diagnosis, Glasgow Coma Scale (GCS), length of stay in ICU (LoS), time between admission and development of the pneumonia, days of mechanical ventilation (VM) and presence of hypotension (defined as arterial systolic pressure less than 90 mmHg despite adequate fluid resuscitation in the emergency department). CXR, body temperature, leukocyte count, creatinine, presence of sputum and results of mini-BAL, in particular, were obtained at 72h, at 7 days, at 28 days or until discharge. The outcome at 28 days or at discharge was classified as: healing, microbiological eradication or persistence and exitus.

### VAP diagnosis and etiology

Diagnosis of Ventilator Associated Pneumonia (VAP) was defined as pneumonia that occurred ⩾48 hours after hospital admission and, according to international guidelines of the CDC in Atlanta, it was diagnosed by the finding of a new or progressive pulmonary infiltrate associated with at least two of the following criteria: fever > 38°C, leukocytosis > 11,000 cells/mm^3^ or leucopenia < 4.000 cells/mm^3^, purulent respiratory secretions [25]. VAP was defined as nosocomial pneumonia in a patient with at least 48 hours of mechanical ventilator support. VAP was defined as “*early onset*” or “*late onset*” depending on whether the infection occurred within the first 4 days of VM or later.

The etiology of the pneumonia was established by isolation of the organism from blood cultures or by culturing the organism from endotracheal aspirates or mini-BALs. By etiology, VAP were divided into two groups: VAP caused by *Acinetobacter baumanii* (AbVAP group) and VAP caused by other microorganisms (VAP group). We have compared these two groups regarding: mortality, eradication, LoS and days of VM.

### Colistin

We reported antibiotic therapy, its duration, the posology and the possible reasons for the precocious termination of therapy. In particular, we have studied the data relating to the Colistin. It was used as salvage therapy in infections due to *Acinetobacter baumanii* carbapenem-resistant (XDR). The dosages used were 3mUI of MSM (Colistinmetate sodium) every 8 hours i.v..

Renal function was monitored by daily measurement of the serum creatinine level.

### Statistical Analysis

Statistical analysis between AbVAP group and VAP group was performed by an independent sample t test and exact Fisher test. P values<0.05 were considered statistically significant.

## III. RESULTS

From January 2011 through January 2012, 338 patients were hospitalized in Intensive Care Unit of AOU Federico II University. A total of 22 patients (6,51%) developed nosocomial respiratory infections.

All patients received mechanical ventilation (mean length was 14,6 ± 3,75 days).

18 (82% of 22 patients) episodes were classified as VAP. Out of 18 patients investigated, 10 (56%) were male and 8 (44%) were female.

The mean age was 47,8 ± 21,7 years for AbVAP group and 69 ± 16,7 years for the VAP group.

Ten (56%) episodes of VAP were due to Acinetobacter spp. and eight (44%) to different etiology (including *P. Aeruginosa* and *Klesbiella pneumoniae*). Five patients (50%) belonging to the AbVAP group developed *early onset* pneumonia. Conversely, with regard to the VAP group, *early onset* pneumonia arose in only two patients (25%).

Severity of illness upon admission to the ICU was similar between the two groups. More in details, the SOFA score was 4 ± 1,89 in AbVAP group vs. 4,7 ± 2,6 in VAP group. The SAPSII score was 17,6 ± 6,36 in AbVAP group vs. 21,5 ± 11,5 in VAP group.

The most notable finding was the SOFA score at diagnosis (3.7 ± 1.4 in AbVAP group vs. 6.9 ± 3.6 in VAP group; p<0.05).

Risk factors incidence was similar: hypertension was 50% in AbVAP group vs. 80% in VAP group; diabetes had an impact of 20% in the first group and 50% in the second group. The mean duration of stay in ICU was 32,3 ± 17,4 (AbVAP group) and 37,8 ± 14,5 (VAP group).

The 28-day mortality was corresponding in both units (20% AbVAP vs. 30% VAP). The treatment was based on Colistin (CMS= 3mUIx3/die); the mean duration of therapy was 14.6 ± 3.75 days; the frequency of eradication was 6/10 in the group AbVAP and 6/8 in VAP group (not statistically significant).

Premature end of therapy for renal failure cases was not observed.

Data are presented in Table 1.

## IV. DISCUSSION

VAP is the most common infection in intensive care units (ICU); it contributes significantly to the morbidity and mortality of ICU patients, with an estimated incidence rate of 8% to 28%.

In a previous review published by American Journal of Respiratory and Critical Care Medicine, Castre et al reported that more than 60% of VAP is caused by aerobic GNB. The predominant GNB were *P. aeruginosa* and *Acinetobacter* spp., followed by *Proteus* spp., *Escherichia coli, Klebsiella* spp., and *H. influenzae*. As in other studies, we found that the incidence of VAP is 11% and 60% of cases is caused by *Acinetobacter baumanii*[5].

Knowledge of predictors for the isolation a certain pathogen in patients with the clinical diagnosis of VAP has important practical implications since it determines which patients should receive active antimicrobials against this organism. Previous studies identified several factors associated with the isolation of *A. baumannii* in respiratory samples of patients with lung infection: prior sepsis, previous antibiotic use, re-intubation, length of hospital stay before infection, length of mechanical ventilation before diagnosis, exposure to imipenem, and exposure to fluoroquinolones [26,27,28].

Despite somewhat different definitions of early-onset pneumonia, varying from <3 to <7 days [28,29,30], drug-resistant pathogens were more frequently observed in the late-onset cases than in the early-onset cases. Previous studies have reported that late-onset VAP was more often caused by *Pseudomonas aeruginosa*, *Acinetobacter species*, methicillin-resistant *Staphylococcus aureus*, vancomycin-resistant enterococci, extended-spectrum b-lactamase–producers, and multiresistant Gram-negative bacilli, whereas early-onset VAP was more often caused by *Haemophilus influenzae*, *Streptococcus pneumoniae*, methicillin-susceptible *S. aureus*, and Enterobacteriaceae [28]. Antibiotic use is one of the major determinants of the shift toward resistant strains [26].

Caricato et al in a retrospective analysis performed at the intensive care unit of “A Gemelli Hospital” located in Rome, Italy, found that all *Acinetobacter b*. related pneumonia were “late onset”[31].

Trouillet et al. identified several factors related to the acquisition of VAP due to MDR microorganisms: 7days- mechanical ventilation, prior antimicrobial therapy and the use of broad-spectrum antibiotics [32].

Similarly, Rios et al. identified hospitalization prior to VAP onset during 5 days and the use of prior antimicrobial therapy as risk predictors of VAP caused by MDR pathogens [33].

In our study we found a VAP incidence of 5% (18/338); 10 out of 18 (56%) episodes of VAP were due to Acinetobacter spp. and eight (44%) to different etiology (including *P. Aeruginosa* and *Klesbiella pneumoniae*). Five patients (50%) belonging to the AbVAP group developed *early onset* pneumonia. Conversely, with regard to the VAP group, *early onset* pneumonia arose in only two patients (25%). In addiction we not found different distribution pattern of etiologic agents between early- and late-onset VAP. Notably we found particularly high rates of early onset AbVAP (5/10) but we think that this data are attributable to a misrecognized outbreak of *A. baumannii*. More in details 3 out of 5 episodes are temporally close.

A number of variables have been identified as independent predictors of outcome of pneumonia including severity of illness, presence of organ dysfunction or failure, underlying diseases and comorbidities, and specific causative pathogens (MDR pathogens or not) [28,34].

Previous studies have identified severity of illness as risk factor for VAP. Different prognostic scoring systems are used in critical care, including the Therapeutic Intervention Scoring System, the Mortality Predictor Model, the Simplified Acute Physiology Score, and the APACHE II, APACHE III, and APACHE IV scores. The value of the APACHE II, SOFA and CPIS scores in the prediction of mortality during VAP episodes was assessed.

Gupta et al [28] in a prospective cohort study of 107 patients observed that APACHE II score was an important parameter to stratify risk of developing VAP. A significantly higher value was observed in VAP group (P < 0.001).

Garnacho-Montero et al. found that the severity of illness at the moment of pneumonia diagnosis measured by the SOFA scale is an independent predictor of fatality. Notably in the same study the autors have reported that the two groups of patients whose pneumonia was or was not caused by *A. baumannii* were similar with regard to severity of illness at admission measured by the APACHE II score [26].

We observed that the two arms of study were similar with regard severity of illness at admission measured by SAPSII score and SOFA score. However, we found the SOFA score determined at the time of VAP diagnosis to be increased among patients belong to VAP group. We believe that our data do not conflict with above-mentioned studies because if scoring systems for severity of illness may be useful in stratify the risk of developing VAP, they do not have to correctly identify an AbVAP.

The comorbidity analysis in our work is consistent with data obtained in other reports. We suggest that patients acquiring AbVAP are no more comorbid than patients that have VAP. Similarly, in terms of comorbidities Garnacho-Montero et al not found significant difference between patients with and without AbVAP [26]. Likewise, Gupta et al observed that the chronic illnesses were not more prevalent in VAP patients [28].

The most notable finding of the present study is that mortality rate does not differ between AbVAP group and VAP group.

Should be emphasized that there is debate in the literature on the impact of *Acinetobacter* species infection on crude and attributable mortality. Intuitively, one could suppose that a pulmonary infection caused by multidrug resistant *A. baumannii* increases mortality. However, many variables are involved in the clinical course of critically ill patients.

Fagon et al in a case-control study, for example, reported significantly higher mortality in cases of VAP caused by *P. aeruginosa* or *A. baumannii*[35].

Similarly, Kollef et al. found that VAP due to non-fermentative Gram-negative pathogens was an independent predictor of in-hospital mortality [36].

On the contrary, Garnacho-Montero used two different methods (case-control study and multivariate analysis) to demonstrate that episodes of pneumonia caused by multidrug resistant *A. baumannii* do not carry a worse prognosis than other causes of pulmonary infection in ventilated patients [26].

This finding is in agreement with a case-control study which concluded that VAP caused by *A. baumannii* was not associated with an increase in the risk of death, although imipenem-resistant episodes were associated with a tendency to higher mortality rates [37].

There are various explanations for these apparently controversial findings: first, unspecific risk factors may not have been included in the matching process of case–control and cohort studies (and thus differences in mortality may be attributed to these particular factors).

Secondly, there are great differences in patient populations, methodological characteristics of the studies, proportions of patients who received appropriate empirical treatment, as well as factors associated with the pathogen itself, including genetic factors that lead to differences in virulence.

At present, Colistin is rescue therapy for AbVAP.

Potential therapeutic indications for Colistin have been restored and a number of studies have assessed its use in ICU with most commonly used regimens of 2MUI, 3MUI of CMS (Colistinmetate sodium) every 8 hours or 2.5–5mg of Colistin base divided into 3 doses/die [38].

For example, Levin et al in a case series examined 59 patients infected with *P. aeruginosae* and *A. baumanii*. Good clinical outcomes was obtained in 58% of cases; although the author found that the poorest results were observed in cases of pneumonia, compared with other types of infections [39].

Garnacho-Montero et al evaluated patients with VAP due to *A. Baumanii*, with 21 episodes caused by microorganism susceptible exclusively to Colistin and 14 susceptible to imipenem. The clinical cure, defined as remission of pneumonia symptoms was high (57%) [40]. Furthermore, in a study of 185 critically ill patients with *Acinetobacter* spp. or *P. aeruginosa* infections, 55 were treated with Colistin and 105 of the remaining 130 were treated with a carbapenem. Accordingly Reina et al. concluded that Colistin was as safe and effective as other antibiotics [41].

Finally, in a retrospective study, Rios et al compared 31 patients with VAP caused by MDR *Acinetobacter* spp. or *P. aeruginosa* that preserve their susceptibility to carbapenems with 30 patients affected by VAP sustained by only-Colistin susceptible strains; he found that intravenous Colistin is effective [33].

In our study all patients in AbVAP arm were treated with Colistin and the clinical resolution rate is 80%. Despite this finding, we observed a low bacteriological eradication rate (60%). These data are consistent with data reported by Garnacho-Montero in above-mentioned studies [40,26].

However, there are limited information on the pharmacokinetics of Colistin intravenous administration and its effectiveness for treatment of pneumonia because of its inadequate penetration in the lung parenchyma [38]. With regard to the toxicity related to the use of Colistin we did not observe an increase of renal failure related to therapy.

Considering the high mortality of VAP and low bacteriological eradication rate, nebulized Colistin has been employed as an attempt to minimize systemic toxicity and improve drug deposition at the site of infection.

Much of the data on inhaled Colistin originates from patients with cystic fibrosis (CF) who are colonized or infected with *Pseudomonas aeruginosa*. However, the literature is expanding outside this patient population.

In a retrospective case series by Kwa et al., 18 of 21 (86%) patients who received nebulized Colistin for hospital-acquired pneumonia caused by *A. baumannii* or *P. aeruginosa* (resistant to all antimicrobials except polymyxins) had favorable clinical and microbiological responses [42].

Similar, encouraging results were reported by Michalopoulos et al., who compared the outcomes for 8 patients who received supplemental nebulized Colistin combined with parenteral antibiotics with those for 45 patients who received parenteral Colistin only. Rates of clinical cure appeared superior with supplemental nebulized Colistin, but the patient numbers were small thus limiting statistical comparisons [43,44,45].

However, neither Food and Drug Administration (FDA) nor European Medicines Agency (EMEA) approved the use of inhaled Colistin form in non CF-Patients. Literature about Colistin efficacy is summarized in table 2.

We acknowledge that our study has some limitations, since it is a retrospective and mono-center analysis and the sample is relatively small. We cannot overlook the impact of the architectural design of the ICUs on the transmission of *A. baumannii*. Third, it is well known that the incidence of multidrug resistant pathogens is closely linked to local factors and varies generally from one institution to another.

## V. CONCLUSION

VAP episodes caused by *A. baumannii* have a similar comorbidity, prognostic scores, mortality and eradication frequency to pneumonia episodes caused by other virulent pathogens.

Colistin is a valid option for treating episodes of VAP due to carbapenem resistant strains and it should be used as first-line therapy for *A. baumannii* VAP if local epidemiological data show a high incidence for MDR strains.

Even if in our study Colistin has 80% of healing, it is associated to 60% eradicated VAP episodes only.

The pharmacokinetic-pharmacodynamics relationship of this antibiotic should be investigated further, in particular for inhalation use. In fact formulations for inhalation therapy are limited and minimal data support the high cost of such therapies. More data on efficacy and safety of inhaled antimicrobial therapy in specified populations of patients with VAP are needed before such therapy could be recommended for routine clinical use.

With regard to the toxicity related to the use of Colistin we did not observe an increase of renal failure related to therapy.

Waiting for definitive data on Colistin and in the absence of new active drugs, strict application of prevention rule, as specified in the European HAP guidelines, is the only possibility to reduce the AbVAP mortality.

## Figures and Tables

**TABLE 1:** 

	**Patient features**		
	
	AbVAP	VAP	Statistical analysis

Age	47,8±21,7	69±16,7	P <0,05
M	4	6	
F	6	2	
Weight	87,5±30,2	80±10	NS
SOFA dx	3,7±1,4	6,9±3,6	P <0,05
SOFA admission	4±1,89	4,7±2,6	NS
SAPS II admission	17,6±6,36	21,5±11,5	NS
Hyertension	50%	80%	NS
Diabetes	20%	50%	NS
	
	**Outcomes**		

Mortality	20%	30%	NS
Eradication	60%	75%	NS
Length VM	20,9±8,49	23,1±11,15	NS
LoS	32,3±17,5	37±14,5	NS

**TABLE 2:** THE COLISTIN EFFICACY

References	Report features				
	Number of patiens	Conditions treated	Pathogen	Therapy	% success
Reina (40)	55	VAP+ other infection	P. aeruginosa (35%) A. Baumanii (65%)	5 mg/kg per day divided into three doses iv	66%
Levin (38)	59	Pneumonia + other infection	P. aeruginosa (35%) A. baumanii (65%)	6,67–13,3mg/Kg per day divided into three doses iv	58% (25% for pneumonia)
Garnacho_ (39)	21	VAP	P. aeruginosa (35%) A. baumanii (65%)	2,5–5mg/kg every 8h iv	57%
Kwa (41)	21	Pneumonia (3VAP)	P. aeruginosa (19%) A. baumanii (81%)	1 million units of inhaled Colistin every 8h+other antimicrobials iv	85,7%
Michalopoulos (43)	8	VAP	P. aeruginosa (13%) A. baumanii (87%)	0,5–2million units of inhaled Colistin every 8h+2million units Colistin every 8h o other antimicrobials iv	88%
Michalopoulos (44)	60	VAP	P. aeruginosa (20%) A. baumanii (62%) K. pneumoniae (18%)	2.2(±0.7) million international units of inhaled Colistin + Colistin o other antimicrobials iv	83%

## References

[1] Piazza O, Tufano R, Papaianni C (2008). Le infezioni nosocomiali in terapia intensiva.

[2] National Nosocomial Infections Surveillance (NNIS) (2004). System report, data summary from January 1992 through June 2004, issued October 2004. Am J. Infect Control.

[3] Health Protection Agency Mandatory Surveillance: Mandatory Staphylococcus aureus bacteraemia surveillance scheme. http://www.hpa.org.uk/infections/topics_az/staphylo/staphylo_mandatory_surveillance.htm.

[4] Malacarne (2010). “Epidemiology of nosocomial infection in 125 Italian intensive care”. Minerva Anestesiologica.

[5] Chastre J (2002). Ventilator-associated pneumonia. Am J Respir Crit Care Med.

[6] Jawad A (1998). Exceptional desiccation tolerance of Acinetobacter radioresistens. J Hosp Infect.

[7] Catalano M (1999). Survival of Acinetobacter baumannii on bed rails during an outbreak and during sporadic cases. J Hosp Infect.

[8] Van den Broek PJ Epidemiology of multiple Acinetobacter outbreaks in the Netherlands during the period 1999–2001. Clin Microbiol Infect.

[9] Dijkshoorn L (2007). An increasing threat in hospitals: multidrugresistant Acinetobacter baumannii. Nat Rev Microbiol.

[10] Van Looveren M, ARPAC Steering Group (2004). Antimicrobial resistance of Acinetobacter spp. in Europe. Clin Microbiol Infect.

[11] Dijkshoorn L (1996). Comparsion of outbreak and nonoutbreak Acinetobacter baumannii strains and phenotypic methods. J Clin Microbiol.

[12] Coelho JM (2006). Occurrence of carbapenem-resistant Acinetobacter baumannii clones at multiple hospitals in London and Southeast England. J. Clin Microbiol.

[13] Nemec (2008). Emergence of carbapenem resistance in Acinetobacter baumannii in the Czech Republic is associated with the spread of multidrug resistant strains of European clone II. J Antimicrob Chemother.

[14] Zarrilli R (2007). Molecular epidemiology of a clonal outbreak of multidrug-resistant Acinetobacter baumannii in a university hospital in Italy. Clin.Microbiol. Infect.

[15] Gaynes R (2005). Overview of nosocomial infections caused by Gram-negativebacilli. Clin Infect Dis.

[16] Wisplinghoff H (2004). Nosocomial bloodstream infections in US hospitals analysis of 24.179 cases from a prospective nationwide surveillance study. Clin Infect, Dis.

[17] Ji Ye Jung (2010). Risk factors for multidrug resistant Acinetobacter baumanii bacteriemia in patients with colonization in the intensive care unit. BMC Infectious disease.

[18] Wisplinghoff H (1999). Risk factors for nosocomial bloodstream infections due to Acinetobacter baumanii: a case control study of adult burn patients. Clin Infect Dis.

[19] Johnson EN (2007). Infectious complications of open type III tibial fractures among combat casualties. Clin Infect Dis.

[20] Metan G (2007). Acinetobacter baumanii meningitis in post neuro surgical patients: clinical outcame and impact of carbapenem resistance. J. Antimicrob. Chemiother.

[21] Michalopoulos AS (2005). Colistin treatment in patients with ICU acquired infections caused by multiresistant Gram-negative bacteria: the renaissance of an old antibiotic. Clin Microbiol. Infect.

[22] Landman D (2008). Polymyxins Revisited Clinical Microbiology Reviews.

[23] Moffatt JH (2010). Colistin resitance in Acinetobacter baumanii is mediated by complete loss of lipopolycaccharide production. Antimicrobial Agents and Chemiotherapy.

[24] Lopez-Rojas R

[25] (1997). “Guidelines for prevention of nosocomial pneumonia”. Centers for Disease Conrol and Prevention. MMWR.

[26] Garnacho-Montero (2005). Acinetobacter baumanii ventilator associated pneumonia: epidemiological and clinical findings. Intensive Care Med.

[27] Chang HC (2011). Mortality risk factors in patients with Acinetobacter baumanii ventilator-associated pneumonia. J of Formosan medical Association.

[28] Gupta (2011). Incidence, rick stratification, antiobiogram of pathogens isolated and clinicl outcome of ventilator associated pneumonia. Indian J Crit Care Med.

[29] Langer M (1989). Long-term respiratory support and risk of pneumonia in critically ill patients. Intensive Care Unit Group of Infection Control. Am Rev Respir Dis.

[30] Langer M (1987). Early onset pneumonia: a multicenter study in intensive care units. Intensive CareMed.

[31] Caricato (2009). Risk factors and outcome of Acinetobacter baumanii infection in severe trauma patients. Intensive Care Med.

[32] Trouillet JL, Chastre J, Vaugnat A (1998). Ventilator associated pneumonia caused by potentially drug-resistant bacteria. Am J Respir Crit Care Med.

[33] Rios (2002). Multiresistant pathogens and antimicrobial prescribing practice for ventilator associated pneumonia. Impact of prior duration of mechanical ventilation or stay up and antibiotics use. CHEST.

[34] Ebru Cakir Edis (2011). Acinetobacter pneumonia: is the outcome different from the pneumonias caused by other agents. Ann Thorac Med.

[35] Fagon (1993). Nosocomial pneumonia in ventilated patients: a cohort study evaluated attributable mortality and hospital stay. Am J Med.

[36] Kollef (1997). The impact of nosocomial infections on the patients outcome following cardiac surgery. CHEST.

[37] Garnacho J (2003). Clinical impact of pneumonia caused by Acinetobacter baumannii in intubated patients:A matched cohort study. Crit Care Med.

[38] Imberti R (2010). Steady- state pharmacokinetics and BAL concentration of Colistin in critically ill patients after IV colistin Methasulfonate administration. CHEST.

[39] Levin (1999). Intravenous Colistin as therapy for nosocomial infections caused by multidrug-resistant Pseudomonas aeruginosa and Acinetobacter baumanii. Clin Infect Dis.

[40] Garnacho-Montero J (2003). Treatment of multidrug resistant Acinetobacter baumanii ventilator associated pneumonia(VAP) with intravenous Colistin: a comparison with Imipenem-susceptibile VAP. Clin Infect Dis.

[41] Reina (2005). Safety and efficacy of Colistin in Acinetobacter and Pseudomonas infections: a prospective cohort study. Intensive Care Med.

[42] Kwa ALH (2005). Nebulized Colistin in the Treatment of Pneumonia Due to Multidrug-Resistant Acinetobacter baumannii and Pseudomonas aeruginosa. Clinical Infectious Diseases.

[43] Michalopoulos A (2011). Aerosol delivery of antimicrobials agents during mechanical ventilation: current practice and perspectives. Curr Drug Delivery.

[44] Michalopoulos A (2005). Aerosolized Colistin for the treatment of nosocomial pneumonia due to multidrug-resistant gram negative bacteria in patients witouth cyctic fibrosis. Crit Care.

[45] Michalopoulos A (2008). Aerosolized Colistin as adjuntive treatment of ventilator-associated pneumonia due to multidrug-resistant gram negative bacteria : a perspective study. Resp Med.

